# Association of Cumulative Social Risk and Social Support With Receipt of Chemotherapy Among Patients With Advanced Colorectal Cancer

**DOI:** 10.1001/jamanetworkopen.2021.13533

**Published:** 2021-06-09

**Authors:** Rachel E. Davis, Amber W. Trickey, Paul Abrahamse, Ikuko Kato, Kevin Ward, Arden M. Morris

**Affiliations:** 1 Department of Health Promotion, Education, and Behavior, Arnold School of Public Health, University of South Carolina, Columbia; 2 Stanford–Surgery Policy Improvement Research and Education Center, Department of Surgery, Stanford University School of Medicine, Stanford, California; 3 Department of Biostatistics, University of Michigan, Ann Arbor; 4 Department of Pathology, Karmanos Cancer Institute, Wayne State University, Michigan; 5 Department of Epidemiology, Rollins School of Public Health, Emory University, Atlanta, Georgia

## Abstract

**Importance:**

Approximately 38% of patients with advanced colorectal cancer do not receive chemotherapy.

**Objective:**

To determine whether cumulative social risk (ie, multiple co-occurring sociodemographic risk factors) is associated with lower receipt of chemotherapy among patients with advanced colorectal cancer and whether social support would moderate this association.

**Design, Setting, and Participants:**

This cross-sectional, population-based, mailed survey study was conducted from 2012 to 2014. Participants were recruited between 2011 and 2014 from all adults within 1 year after diagnosis of stage III colorectal cancer in the Detroit, Michigan, and State of Georgia Surveillance, Epidemiology, End-Results cancer registries. Patients were eligible if they were aged 18 years or older, had undergone surgery 4 or more months ago, did not have stage IV cancer, and resided in the registry catchment areas. Data analyses were conducted from March 2017 to April 2021.

**Main Outcomes and Measures:**

The primary outcome was receipt of chemotherapy. Cumulative social risk represented a sum of 8 risk factors with the potential to drain resources from participants’ cancer treatment (marital status, employment, annual income, health insurance, comorbidities, health literacy, adult caregiving, and perceived discrimination). Social support was operationalized as emotional support related to colorectal cancer diagnosis.

**Results:**

Surveys were mailed to 1909 eligible patients; 1301 completed the survey (response rate, 68%). A total of 1087 participants with complete data for key variables were included in the sample (503 women [46%]; mean [SD] age, 64 [13] years). Participants with 3 or more risk factors were less likely to receive chemotherapy than participants with 0 risk factors (3 factors, odds ratio [OR], 0.48 [95% CI, 0.26-0.87]; 4 factors, OR, 0.41 [95% CI, 0.21-0.78]; 5 factors, OR, 0.42 [95% CI, 0.20-0.87]; ≥6 factors, OR, 0.22 [95% CI, 0.09-0.55]). Participants with 2 or more support sources had higher odds of undergoing chemotherapy than those without social support (2 sources, OR, 3.05 [95% CI, 1.36-6.85]; 3 sources, OR, 3.24 [95% CI, 1.48-7.08]; 4 sources, OR, 3.69 [95% CI, 1.71-7.97]; 5 sources, OR, 4.40 [95% CI, 1.98-9.75]; ≥6 sources, OR 5.95 [95% CI, 2.58-13.74]). Within each social support level, participants were less likely to receive chemotherapy as cumulative social risk increased.

**Conclusions and Relevance:**

Cumulative social risk was associated with reduced receipt of chemotherapy. These associations were mitigated by social support. Assessing cumulative social risk may identify patients with colorectal cancer who are at higher risk for omitting chemotherapy who can be targeted for support programs to address social disadvantage and increase social support.

## Key Points

**Question:**

Is cumulative social risk (ie, co-occurring sociodemographic barriers) associated with lower receipt of chemotherapy among patients with advanced colorectal cancer, and does social support moderate this association?

**Findings:**

Data from a cross-sectional survey of 1087 diverse adults with stage III colorectal cancer indicated that participants with 3 or more social risk factors were less likely to receive chemotherapy than participants with 0 risk factors. The association of cumulative social risk with chemotherapy receipt was moderated by access to social support.

**Meaning:**

These findings suggest that assessing cumulative social risk may identify patients with advanced colorectal cancer who are at higher risk for omitting chemotherapy.

## Teaser

This survey study examines whether cumulative social risk is associated with lower receipt of chemotherapy among patients with advanced colorectal cancer (CRC) and whether social support moderates this association.

## Introduction

Adjuvant chemotherapy after surgery among patients with stage III colorectal cancer (CRC) is associated with up to a 30% increase in 5-year survival rates.^1,2,3^ Yet, among the 40 000 US individuals with recent diagnoses of stage III CRC,^4^ approximately 38% will not receive adjuvant chemotherapy, with no discernable clinical rationale.^5,6,7,8^ Patients need resources to accommodate the physical, financial, cognitive, and emotional demands of chemotherapy, and health, demographic, and social factors may deter chemotherapy initiation among patients with CRC. Older patients with extensive comorbid disease are more likely to experience delays or omission of chemotherapy, even absent clinical concerns. With few exceptions,^9,10^ studies have found lower rates of chemotherapy receipt among Black patients compared with White patients with CRC, perhaps because of economic and social disadvantage.^6,11,12,13,14,15,16^ Patients with lower income or health literacy levels are also less likely to receive chemotherapy.^11,17,18,19,20^ The association of perceived discrimination, owing to race or other characteristics, with chemotherapy or other treatment uptake are unknown, but previous studies suggest that there is an association between discrimination and reduced CRC screening.^21,22^ To our knowledge, no preexisting research has examined the cumulative associations of multiple, co-occurring social risk factors with chemotherapy receipt for CRC, which may serve as more powerful deterrents than a single barrier alone.

In contrast, social support appears to have a beneficial association with chemotherapy receipt. Married patients with CRC have higher rates of chemotherapy receipt,^11,13,15^ fewer delays in starting chemotherapy,^19^ and greater likelihood of completing chemotherapy than single adults.^15^ Studies outside the US indicate that social support is associated with stress reduction during chemotherapy,^23^ completion of chemotherapy,^24^ and overall and CRC-specific survival.^25^ More research is needed to examine the associations of social support with chemotherapy receipt among patients with CRC in the US.

This survey study sought to examine whether cumulative social risk was associated with lower use of chemotherapy among patients with advanced CRC. On the basis of the buffering model, which predicts that the adverse effects of stress may be reduced by social support,^26^ we further hypothesized that social support would mitigate the association between cumulative social risk and chemotherapy receipt.

## Methods

The study protocol was approved by institutional review boards at the University of Michigan, Wayne State University, Emory University, the State of Michigan, and the State of Georgia. In keeping with study participant deidentification protocols, all relevant institutional review boards granted a waiver of written informed consent. This study follows the American Association for Public Opinion Research (AAPOR) reporting guideline.

### Questionnaire Development

The questionnaire was specifically developed to focus on experiences with CRC and was based on well-described conceptual frameworks, including the Mandelblatt^27^ and Andersen^28^ models of equity in access to and use of care. Through an extensive literature review, we identified and included existing, validated instruments that captured the relevant domains whenever possible. As appropriate, these instruments were modified to refer specifically to experiences with the diagnosis and treatment of CRC. When preexisting measures were unavailable, the study team generated questions based on preliminary focus group data. A nearly final version of the questionnaire was cognitively pretested among patients who would have been eligible to participate but were not included. On the basis of their responses, we iteratively modified and repeat tested the survey until the survey items and respondent burden stabilized.

### Participants

All eligible adults who had received a diagnosis of stage III CRC within 1 year, between August 2011 and December 2014, were identified in the Detroit, Michigan, tri-county area and the State of Georgia Surveillance, Epidemiology, End-Results (SEER) cancer registries using Rapid Case Ascertainment. Patients were eligible if they were aged 18 years or older, had undergone surgery at least 4 months ago, did not have stage IV cancer, and resided in the targeted SEER catchment areas.

### Data Collection

After allowing physician opt-out, patients were invited to participate in a self-administered mailed survey, which included a $10 preincentive and up to 9 multimodal contact attempts.^29^ Completed questionnaires were accepted up to 1 year after surgery. Participants were recontacted, as necessary, to clarify responses or obtain missing information. The overall survey response rate was calculated in accordance with American Association for Public Opinion Research^30^ standards as the number of unique surveys that were completed and returned by eligible patients divided by the total number of eligible patients. The total number of eligible patients was the sum of those who returned surveys, refused participation, could not be located, or were prohibited from participation by the physician of record.

### Measures

#### Receipt of Chemotherapy

The standard of care for treatment of stage III CRC during the study period included initiation of chemotherapy within 4 months after surgery. Receipt of chemotherapy was defined as a yes response to the question, “Did you or are you going to have chemotherapy to treat your colorectal cancer?” and a yes or “I’m still receiving chemotherapy treatment” response to the question, “Did you have ALL of the chemotherapy treatments that were first planned?” Nonreceipt of chemotherapy was defined a no or “I have not started chemotherapy treatment” response, indicating no initiation of chemotherapy within 4 months of surgery. Initiation of chemotherapy more than 4 months postoperatively no longer confers a survival advantage for stage III CRC and is no longer recommended.^31,32^

#### Cumulative Social Risk

Cumulative social risk represented positive responses to questions assessing 8 social risk factors with the potential to drain time, energy, financial, or other resources from cancer treatment. In the interests of clarity and the absence of literature to guide assigning weights to social risk, we decided a priori to dichotomize. Accordingly, each social risk factor was assigned a value of 1, indicating the presence of higher risk, and a value of 0, indicating lower risk, with a summative range of 0 to 8. However, because no participants reported 8 factors and only 11 participants reported 7 factors, the 6 to 8 categories were collapsed, yielding a final variable with values ranging from 0 to 6or more, with higher scores indicating increased cumulative social risk. The following risk factors were included: marital status, employment, income, health insurance, comorbidities, health literacy, adult caregiving, and perceived discrimination.

For marital status, participants who were married or living with a partner were assigned a risk value of 0. All others were assigned a 1.

For employment, participants who were unemployed or disabled at the time of the survey were assigned a 1. All others were assigned a 0.

For income, participants reported their annual household income at diagnosis (<$20 000, $20 000-$49 999, $50 000-$89 999, and ≥$90 000), with multiple imputation used to estimate income from respondents’ demographic data (age, sex, race, education, and marital status) for 246 respondents with missing data. Participants with an annual income of $50 000 or higher, or approximately twice the Federal Poverty Level for a family of 4, were assigned a 0, whereas all others were assigned a 1.

For health insurance, participants with Medicaid or no insurance at diagnosis were assigned a 1, and participants with Medicare, private insurance, or other insurance were assigned a 0. If data were missing, responses to an item querying employer-provided benefits at diagnosis were reviewed, and participants with health insurance benefits were coded as 0. If responses to both items were missing, the risk score was based on a question assessing insurance status at the time of the survey.

For comorbidities, 10 items queried whether participants had been told by a doctor that they had chronic bronchitis or emphysema, heart disease, cancer (not skin cancer or CRC), diabetes, gastrointestinal problems (eg, irritable bowel syndrome), high blood pressure, stroke, liver disease; kidney failure, or depression. On the basis of previous work from our group and others^33,34^ indicating progressively diminishing association with outcomes when adjusting for 2 or more comorbidities, participants were assigned a 1 if they reported 2 or more comorbidities and a 0 for 1 or fewer comorbidities.

Health literacy was represented by the mean of 3 items previously validated in clinical populations,^35,36^ which were slightly adapted to focus on difficulties understanding their CRC or CRC treatment. Participants who said they never or occasionally had difficulty on all 3 items were categorized as having adequate health literacy and assigned 0. All others were categorized as having inadequate or marginal health literacy and were assigned a 1.

With regard to adult caregiving, participants who assisted another adult who lived in their home with personal care were assigned a 1. All others were assigned a 0.

For perceived discrimination, 10 items adapted from the Everyday Discrimination Scale^37,38^ gauged how often participants experienced perceived discrimination in their everyday lives according to age, sex, race/ethnicity, religion, marital status, sexual orientation, weight, income, education, and speech. Participants who had never experienced discrimination were assigned a 0; all others were assigned a 1. This coding was consistent with other research,^39^ acknowledging that even a single experience with discrimination may be traumatic and have long-lasting effects.

#### Social Support

A 7-item scale queried how much emotional support participants had received since diagnosis from 7 sources: spouse or partner, other family members, friends, health care practitioners, coworkers, religious community, or other people with CRC.^40^ Responses of quite a bit or a lot were coded as 1, whereas responses of none, a little, some, or does not apply and missing data were coded as 0. The codes were summed to represent the number of sources of social support. The top 2 categories were collapsed into 6 or more for analysis.

#### Sociodemographic Variables

Single-item measures of sex (male/female), age (25-49, 50-64, and ≥65years), and race (Black and White) were modeled as covariates. Race was included as a covariate because it is independently associated with receipt of chemotherapy. Education was assessed to describe the cohort. Sociodemographic variables were self-identified by participants using the categories provided in the survey instrument.

### Statistical Analysis

Descriptive statistics were calculated for the primary variables. Cumulative social risk and social support were compared by race, sex, and site using 2-sided Wilcoxon rank-sum tests and across age groups using 2-sided Kruskal-Wallis tests. Logistic regression was used to assess the associations of cumulative social risk and social support with chemotherapy receipt.

We first fit a model including cumulative social risk as the primary independent variable while adjusting for age, sex, and race. We then added social support to determine whether associations between chemotherapy receipt and cumulative social risk persisted after adjusting for social support. Finally, to evaluate whether cumulative social risk had a diminished association with chemotherapy treatment when social support was high, we assessed for a moderating association with an interaction term (cumulative social risk × social support) in the logistic regression model using 2 approaches: (1) a continuous interaction between the number of social risk factors and the number of sources of social support, each representing 0 to 6 or more, and (2) a dichotomous 2 × 2 interaction with cumulative social risk and social support dichotomized at the medians. The parsimonious model was then selected. The final model included categorical variables for cumulative social risk and social support to estimate the marginal associations of the number of sources of social support on chemotherapy receipt at each level of cumulative social risk with 95% CIs using Stata/MP statistical software version 14.2 (StataCorp). Statistical significance was set at *P* < .05.

In all sensitivity analyses, we used a decision process guided by the level of missingness in the independent variables. All component social risk variables had missing data for less than 5% of participants, suggesting potentially negligible missing data. Following these practical guidelines, we used the observed data (complete cases) in the main analysis. We did not include respondents who were missing data necessary to calculate the social risk score (110 of 1203 respondents). To estimate the uncertainty due to missingness in these variables, we performed missing data sensitivity analyses as recommended by Jakobsen et al^41^: best-worst case and worst-best case imputation analyses. We found strikingly similar results and model classification performance in the sensitivity analyses, suggesting that the missing data may be ignored (eTable 1 in the Supplement).

To ensure generalizability, we determined variable-specific nonresponse rates using a logistic regression of survey response with those covariates and used the inverse probabilities from that model as the survey weights. We then created additional weights based on these variables to reflect the difference between Rapid Case Ascertainment patients available for survey and the larger population of all patients with stage III CRC in Georgia and Detroit. The final weights are equal to the product of those 2 weighting components and were then standardized so the weighted number equals the unweighted number. We performed sensitivity analyses using the survey weights for the final models (eTable 2 in the Supplement). Data analyses were conducted from March 2017 to April 2021.

## Results

We identified 2168 patients with an incident diagnosis of stage III CRC reported to the SEER registries of Georgia and Detroit using Rapid Case Ascertainment. Among these, 259 (12%) were later determined to be ineligible because they had metastatic disease, noncolorectal primary cancer, a previous cancer diagnosis, or residence outside the registry catchment area. Among 1909 eligible patients included in the final sample, 608 could not be located or did not return the survey, leaving 1301 patients (68% survey response rate). The current study is restricted to the 1087 respondents who provided complete social risk information. The sample was almost half women (503 women [46%]), mostly White (802 participants [74%]), and had a mean [SD] age of 64 [13] years (Table 1). Twenty-two percent of respondents had not received chemotherapy. The mean (SD) number of social risk factors was 2.46 (1.61). The mean (SD) number of sources of social support was 3.97 (1.69).

**Table 1.** Descriptive Statistics for the Study Sample of Patients With Colorectal Cancer

Characteristic	Patients, No. (%) (N = 1087)
Sex	
Female	503 (46)
Male	584 (54)
Age, mean (SD), y	64 (13)
Race	
White	802 (74)
Black	285 (26)
Education	
Less than high school, high school graduate, or general equivalency diploma	442 (41)
Some college or technical school	358 (33)
4-y college graduate or graduate degree	285 (26)
Study site	
Georgia	701 (64)
Detroit, Michigan	386 (36)
Receipt of chemotherapy as intended	845 (78)
Social risk factors	
Marital status	
Married or living with a partner (risk = 0)	655 (60)
Never married, separated, divorced, widowed (risk = 1)	432 (40)
Employment status	
Working part-time, working full-time, homemaker, student, retired (risk = 0)	821 (76)
Unemployed or disabled (risk = 1)	266 (24)
Annual household income, $	
≥50 000 (risk = 0)	473 (44)
<50 000 (risk = 1)	614 (56)
Health insurance	
Private insurance or Medicare (risk = 0)	902 (83)
Medicaid or no insurance (risk = 1)	185 (17)
Comorbidities (in addition to CRC)	
0 or 1 comorbidities (risk = 0)	593 (55)
≥2 comorbidities (risk = 1)	494 (45)
Health literacy	
Adequate (risk = 0)	938 (86)
Marginal or inadequate (risk = 1)	149 (14)
Adult caregiving	
Does not provide caregiving for another adult (risk = 0)	935 (86)
Provides care for at least 1 spouse, parent, parent-in-law, adult relative, or friend who lives in the participant’s home (risk = 1)	152 (14)
Perceived discrimination	
Never (risk = 0)	706 (65)
Rarely, sometimes, often, or very often (risk = 1)	381 (35)
Social risk factors, mean (SD), No.	2.46 (1.61)
Sources of social support, mean (SD), No.	3.97 (1.69)

### Social Risk

Participants who were women (median [interquartile range {IQR}] social risk factors, 3 [2-4]), aged 50 years or older (median [IQR] social risk factors, 2 [1-4] for those aged 50-64 years and 3 [1-3] for those aged ≥65 years), or who identified as Black individuals (median [IQR] social risk factors, 3 [3-4]) had a higher cumulative social risk than those who were younger (median [IQR] social risk factors for those aged 25-49 years, 2 [1-4]), White individuals (median [IQR] social risk factors, 2 [1-3]), or men (median [IQR] social risk factors, 2 [1-3]) (Table 2). Having 1 or 2 risk factors was not associated with reduced chemotherapy receipt (Table 3, model 1); however, participants with 3 (odds ratio [OR] 0.48; 95% CI, 0.26-0.87; *P* = .02), 4 (OR, 0.41; 95% CI, 0.21-0.78; *P* = .007), 5 (OR, 0.42; 95% CI, 0.20-0.87; *P* = .02), or 6 or more (OR, 0.22; 95% CI, 0.09-0.55; *P* = .001) risk factors were significantly less likely to receive chemotherapy than participants with 0 risk factors. Older age (≥65 years) was also associated with lower likelihood of chemotherapy receipt (OR, 0.28; 95% CI, 0.16-0.49; *P* < .001).

**Table 2.** Cumulative Social Risk and Social Support by Sex, Age, Race, and Site

Characteristic	Participants, No. (N = 1087)	Social risk factors, No.			Sources of social support, No.		
		Median (IQR)	Mean (SD)	*P* value^a^	Median (IQR)	Mean (SD)	*P* value
Sex							
Male	584	2 (1-3)	2.27 (1.60)	<.001	4 (3-5)	4.02 (1.70)	.34
Female	503	3 (2-4)	2.68 (1.60)		4 (3-5)	3.91 (1.70)	
Age, y							
25-49	143	2 (1-4)	2.17 (1.90)	.04	5 (3-6)	4.51 (1.60)	<.001
50-64	404	2 (1-4)	2.51 (1.70)		4 (3-5.5)	4.25 (1.60)	
≥65	540	3 (1-3)	2.49 (1.40)		4 (2-5)	3.62 (1.70)	
Race							
White	802	2 (1-3)	2.11 (1.50)	<.001	4 (3-5)	4.04 (1.60)	.04
Black	285	3 (3-4)	3.44 (1.40)		4 (2-5)	3.76 (1.80)	
Site							
Detroit, Michigan	386	3 (1-4)	2.57 (1.60)	.14	4 (3-5)	3.58 (1.70)	<.001
Georgia	701	2 (1-4)	2.40 (1.60)		4 (3-5)	4.19 (1.70)	

Abbreviation: IQR, interquartile range.

^a^ The *P* values in this table were obtained from Wilcoxon rank-sum tests for sex, race, and site, and Kruskal-Wallis tests for age.

**Table 3.** Logistic Regression Estimates of the Association of Cumulative Social Risk and Social Support With Receipt of Chemotherapy

Variable	Participants, No. (N = 1087)	Model 1		Model 2	
		OR (SE) [95% CI]	*P* value	OR (SE) [95% CI]	*P* value
Cumulative social risk					
0	137	1 [Reference]	NA	[Reference]	NA
1	197	1.04 (0.35) [0.54-2.00]	.90	1.06 (0.36) [0.55-2.05]	.87
2	228	0.71 (0.22) [0.39-1.31]	.28	0.79 (0.25) [0.42-1.46]	.44
3	245	0.48 (0.15) [0.26-0.87]	.02	0.56 (0.17) [0.30-1.03]	.06
4	160	0.41 (0.13) [0.21-0.78]	.007	0.50 (0.17) [0.26-0.97]	.04
5	86	0.42 (0.16) [0.20-0.87]	.02	0.54 (0.21) [0.25-1.14]	.11
≥6	34	0.22 (0.10) [0.09-0.55]	.001	0.32 (0.16) [0.12-0.84]	.02
Sources of social support, No.					
0	35	NA	NA	[Reference]	NA
1	61	NA	NA	1.94 (0.87) [0.80-4.68]	.14
2	119	NA	NA	3.05 (1.26) [1.36-6.85]	.007
3	176	NA	NA	3.24 (1.29) [1.48-7.08]	.003
4	260	NA	NA	3.69 (1.45) [1.71-7.97]	.001
5	221	NA	NA	4.40 (1.79) [1.98-9.75]	<.001
≥6	215	NA	NA	5.95 (2.54) [2.58-13.74]	<.001
Race					
Black	285	1 [Reference]	NA	1 [Reference]	NA
White	802	0.84 (0.16) [0.58-1.21]	.34	0.83 (0.16) [0.57-1.20]	.32
Age, y					
25-49	143	1 [Reference]	NA	1 [Reference]	NA
50-64	404	0.97 (0.30) [0.53-1.79]	.93	1.01 (0.32) [0.55-1.86]	.98
≥65	540	0.28 (0.08) [0.16-0.49]	<.001	0.32 (0.09) [0.18-0.57]	<.001
Sex					
Female	503	1 [Reference]	NA	1 [Reference]	NA
Male	584	0.91 (0.14) [0.67-1.24]	.55	0.90 (0.14) [0.66-1.23]	.53
Site					
Georgia	701	1 [Reference]	NA	]1 [Reference]	NA
Detroit, Michigan	386	0.90 (0.14) [0.66-1.22]	.48	1.01 (0.16) [0.74-1.39]	.93
Intercept^a^	NA	15.62 (6.32) [7.07-34.52]	<.001	3.42 (1.88) [1.16-10.04]	.03

Abbreviations: NA, not applicable; OR, odds ratio.

^a^ The intercept represents the log odds of receiving chemotherapy for patients with characteristics at the covariate referent category levels.

### Social Support

Participants reported receiving CRC-related social support from family members other than spouses or partners (939 participants [86%]), followed by friends (842 participants [77%]), health care practitioners (734 participants [68%]), spouses or partners (709 participants [65%]), members of their religious communities (526 participants [48%]), coworkers (331 participants [30%]), and other people with CRC (236 participants [22%]) (eTable 3 in the Supplement). Participants who were younger (median [IQR] sources of social support for those aged 25-49 years, 5 [3-6]) and White participants (median [IQR] sources of social support, 4 [3-5]) reported more social support than those who were older (median [IQR] sources of social support, 4 [3-5.5] for those aged 50-64 years and 4 [2-5] for those aged ≥65 years) and Black participants (median [IQR] sources of social support, 4 [2-5]). Social support was also independently associated with chemotherapy receipt: participants who reported 2 or more sources of social support were more likely to receive chemotherapy than participants with no support (2 sources, OR, 3.05 [95% CI, 1.36-6.85]; 3 sources, OR, 3.24 [95% CI, 1.48-7.08]; 4 sources, OR, 3.69 [95% CI, 1.71-7.97]; 5 sources, OR, 4.40 [95% CI, 1.98-9.75]; ≥6 sources, OR 5.95 [95% CI, 2.58-13.74]).

### Cumulative Social Risk and Social Support

Adding social support to the model reduced the association of cumulative social risk on chemotherapy receipt (Table 3, model 2). Although participants with 4 (OR, 0.50; 95% CI 0.26-0.97; *P* = .04) or 6 or more (OR, 0.32; 95% CI, 0.12-0.84; *P* = .02) risk factors were still less likely than participants with 0 risk factors to undergo chemotherapy, the associations of cumulative social risk were reduced when adjusting for social support. As in model 1, older age was associated with lower likelihood of chemotherapy receipt (age ≥65 years, OR, 0.32; 95% CI, 0.18-0.57; *P* < .001). Interactions between cumulative social risk and social support were not significant when modeled as continuous or dichotomous variables.

Although most participants received chemotherapy, within each level of social support, participants were generally less likely to receive chemotherapy as the number of social risk factors increased (Figure). For example, among those with no social support, the probability of receiving chemotherapy was approximately 60% for participants with 0 risk factors, less than 50% for participants with 3 or more risk factors, and less than 40% for those with 6 or more risk factors. Among participants with 6 or more sources of social support, the probability of receiving chemotherapy decreased from almost 90% for those with 0 risk factors to approximately 75% for those with 6 or more risk factors.

**Figure.**
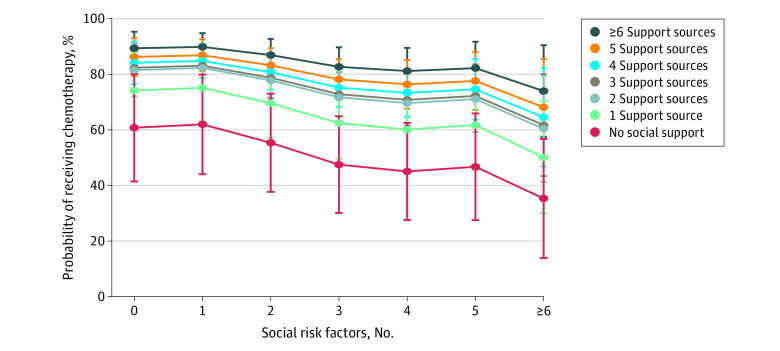
Probability of Receiving Chemotherapy by Cumulative Social Risk and Social Support Data are derived from the logistic regression model 2 for 1087 survey participants with colorectal cancer. Circles indicate means and error bars indicate 95% CIs.

To examine which social risk and social support factors may have been most influential, associations between individual factors and chemotherapy receipt are displayed in Table 4. Marginal or inadequate health literacy (OR, 0.45; 95% CI, 0.31-0.66; *P* < .001), annual income less than $50 000 (OR, 0.55; 95% CI, 0.40-0.77; *P* < .001), single marital status (OR, 0.64; 95% CI, 0.46-0.87; *P* = .005), and perceived discrimination (OR, 0.73; 95% CI, 0.53-0.997; *P* = .048) were each associated with decreased odds of receiving chemotherapy. When we controlled for age, sex, race, and site, additional analyses further indicated that receiving social support from coworkers (OR, 2.60; 95% CI, 1.70-3.98; *P* < .001), health care practitioners (OR, 1.97; 95% CI, 1.45-2.68; *P* < .001), friends (OR, 1.83; 95% CI, 1.32-2.55; *P* < .001), spouses or partners (OR, 1.73; 95% CI, 1.25-2.38; *P* = .001), and family members (OR, 1.63; 95% CI, 1.10-2.41; *P* = .02) were each associated with increased odds of receiving chemotherapy.

**Table 4.** Associations Between Individual Social Risk Factors and Social Support and Receipt of Chemotherapy for 1087 Patients With CRC^a^

Variables	OR (95% CI)	*P* value
Social risk factors		
Health literacy: marginal or inadequate health literacy (reference, adequate)	0.45 (0.31-0.66)	<.001
Annual household income: <$50 000 (reference, ≥$50 000)	0.55 (0.40-0.77)	<.001
Marital status: never married, separated, divorced, or widowed (reference, married or living with a partner)	0.64 (0.46-0.87)	.005
Experiences of everyday discrimination: rarely, sometimes, often, or very often (reference, never)	0.73 (0.53-0.997)	.048
Employment status: unemployed or disabled (reference, working part-time, working full-time, homemaker, student, or retired)	0.80 (0.54-1.20)	.29
Health insurance: Medicaid or no insurance (reference, private insurance or Medicare)	0.86 (0.57-1.30)	.47
Comorbidities (in addition to CRC): ≥2 comorbidities (reference, 0 or 1 comorbidities)	0.86 (0.64-1.17)	.35
Adult caregiving: provides care for at least 1 spouse, parent, parent-in-law, adult relative, or friend who lives in the participant’s home (reference, does not provide caregiving for another adult)	0.94 (0.62-1.43)	.78
Sources of social support^b^		
Coworkers (reference, none, a little, some, does not apply, or missing)	2.60 (1.70-3.98)	<.001
Health care practitioners (reference, none, a little, some, does not apply, or missing)	1.97 (1.45-2.68)	<.001
Friends (reference, none, a little, some, does not apply, or missing)	1.83 (1.32-2.55)	<.001
Spouse or partner (reference, none, a little, some, does not apply, or missing)	1.73 (1.25-2.38)	.001
Family members (excluding spouse or partner) (reference, none, a little, some, does not apply, or missing)	1.63 (1.10-2.41)	.02
Members of religious community (reference, none, a little, some, does not apply, or missing)	1.30 (0.96-1.76)	.09
Other people with CRC (reference, none, a little, some, does not apply, or missing)	1.25 (0.85-1.82)	.26

Abbreviations: CRC, colorectal cancer; OR, odds ratio.

^a^ Results based on multivariable logistic regression models adjusted for age, sex, race, and study site.

^b^ Social support was categorized as high (quite a bit or a lot) vs low (none, a little, some, does not apply, or not reported).

## Discussion

In this population-based survey study, we found that patients with advanced CRC and higher cumulative social risk were less likely than their counterparts with lower social risk to receive adjuvant chemotherapy. Specifically, having 3 or more social risk factors significantly decreased the odds that patients with CRC would receive chemotherapy. The social risk factors most associated with decreased likelihood of chemotherapy receipt included lower health literacy, the lack of a spouse or partner, lower household income, and perceived discrimination. The association of cumulative social risk with chemotherapy receipt was mitigated, however, if patients had strong social support during their CRC treatment. Most participants, regardless of cumulative risk or social support, reported undergoing chemotherapy. However, within each level of social support, we observed a consistent pattern whereby patients with more cumulative social risk were generally less likely to initiate chemotherapy treatment per guideline recommendations.

Previous research on factors associated with chemotherapy receipt among patients with CRC has primarily examined the association of sociodemographic barriers in isolation or in combination with race. However, such analyses may not adequately characterize the complexity of the barriers that socioeconomically vulnerable patients face, because patients from such populations are likely to experience multiple risk factors simultaneously.^42^ Although, to our knowledge, no studies have examined the association of cumulative social risk with receipt of chemotherapy, a study^43^ of Black patients with CRC who had no or inadequate health insurance or who lived in high-poverty neighborhoods found that they were 20% less likely to receive chemotherapy than White patients with CRC with either one of these social risk factors, whereas no racial disparities in chemotherapy receipt were evident among patients with private health insurance living in lower-poverty neighborhoods.

In another study,^44^ no difference was observed in chemotherapy receipt between unmarried and married women with CRC who lived in lower-poverty neighborhoods and had private health insurance. However, among women living in higher-poverty neighborhoods with inadequate health insurance, unmarried women were 26% less likely to receive chemotherapy than married women.^44^ This finding was consistent with our finding that single marital status was associated with decreased chemotherapy receipt.

Among patients without adequate insurance living in high-poverty neighborhoods, chemotherapy receipt was almost 60% lower among Black patients than White patients,^43^ suggesting a multiplicative association of social disadvantage on chemotherapy receipt. These findings are generally consistent with the results of the current study, in that lower income was associated with reduced likelihood of completing chemotherapy and that Black patients with CRC were more likely to have both higher cumulative social risk and fewer sources of social support to mitigate the health associations of cumulative social risk.

Caleyachetty and colleagues^42^ found that higher cumulative social risk exposure (defined as low education, low income, socioeconomically disadvantaged race/ethnicity, and lacking a spouse or partner) was associated with higher all-cancer mortality, and income played a particularly important role. Related research^45,46^ has observed positive associations between other constructions of cumulative social risk and all-cause mortality. These previous studies, in combination with our findings, indicate that although certain social risk factors play a more prominent role, the combined effect of coping with multiple social risk factors concurrently increases the risk that patients with CRC will not undergo chemotherapy treatment as recommended by their health care team.

### Limitations

This study has several limitations, including a reliance on self-reported data and the possibility of recall bias or misremembering timing of events, which may have reduced the validity of data obtained from retrospective questions. We mitigated the potential for inaccurate recall in 3 ways, including references to memorable, highly salient events in the question stem, deploying the survey shortly after diagnosis, and limiting the time span during which returned surveys were accepted. Although it is possible that study respondents made errors when answering these questions, we found the expected distribution of responses and have no reason to believe that the data were adversely impacted by recall bias.

In the absence of previous literature to provide conceptual guidance for our analytical decisions, we weighted social risks equally a priori; however, investigators in future studies of CRC treatment may wish to use the current findings to construct a weighted measure of social risk. We also note that social support was measured using a scale that only assessed emotional support, and, thus, other types of social support (eg, informational, instrumental, and appraisal support) were not reflected in the current analyses. These additional types of social support may be particularly associated with the ability of patients with CRC with high social risk to undergo their CRC treatment as prescribed by their doctors. Although these additional forms of social support were not available in the present data set, they should be included in future research.

Furthermore, the inclusion of stage III colon and rectal cancer from only 2 SEER catchment areas may limit generalizability of our findings. However, the sampling strategy also represents a strength of the study, in that these sites were located in 2 different regions of the US and included a racially diverse sample. We attempted to mitigate nonresponse bias with additional analyses that weighted study responses with SEER site-reported demographic data.

## Conclusions

The findings of this study have at least 4 important clinical implications. First, patients with advanced CRC were less likely to receive chemotherapy as their cumulative social risk increased. This information can be used to identify those patients most at risk for omitting chemotherapy for nonclinical reasons, who can be targeted with patient support programs to address their individualized risk factors, such as help interpreting health information or connecting patients to financial resources. Second, patients with multiple social risk factors may need more holistic patient support programs to undergo their recommended chemotherapy treatment, as opposed to programs that only address isolated social risk factors. Third, social support matters. As in previous research, findings from this study indicated that access to adequate social support minimized the association of social disadvantage with health outcomes. However, this study also found that support from almost any source may be helpful. Although support from family and friends was expected to be beneficial, data from this study indicated that programs to help working patients with CRC obtain support from coworkers may be even more helpful in increasing chemotherapy receipt. Fourth, even for patients with social support, the risk that they would not undergo chemotherapy increased as social risk factors accumulated. Thus, although the availability of social support appeared to be a critical resource for encouraging chemotherapy receipt, patients with multiple social risk factors with social support were still at higher risk of not completing chemotherapy treatment, and, as a consequence, adverse long-term outcomes.

## Notes

Supplement. **eTable 1. **Model Classification Results of Best-Worst and Worst-Best Case Sensitivity Analyses **eTable 2.** Missing Data Proportions and Social Risk Levels of Social Risk Score Component Variables **eTable 3.** Levels of Social Support Perceived From Different Sources (n=1,087)
