# Supplementary material for: Association of Cumulative Social Risk and Social Support With Receipt of Chemotherapy Among Patients With Advanced Colorectal Cancer
Source: JAMA Netw Open. 2021 Jun 9;4(6):e2113533. doi: 10.1001/jamanetworkopen.2021.13533 (PMC8190628; doi:10.1001/jamanetworkopen.2021.13533)
Supplement: Supplement. — eTable 1. Model Classification Results of Best-Worst and Worst-Best Case Sensitivity Analyses eTable 2. Missing Data Proportions and Social Risk Levels of Social Risk Score Component Variables eTable 3. Levels of Social Support Perceived From Different Sources (n=1,087) [file jamanetwopen-e2113533-s001.pdf]

Supplemental Online Content

Davis RE, Trickey AW, Abrahamse P, Kato I, Ward K, Morris AM. Association of cumulative social risk and social support with receipt of chemotherapy among patients with advanced colorectal cancer. *JAMA Netw Open*. 2021;4(6):e2113533. doi:10.1001/jamanetworkopen.2021.13533

eTable 1. Model Classification Results of Best-Worst and Worst-Best Case Sensitivity Analyses
eTable 2. Missing Data Proportions and Social Risk Levels of Social Risk Score Component Variables
eTable 3. Levels of Social Support Perceived From Different Sources (n=1,087)

This supplemental material has been provided by the authors to give readers additional information about their work.

© 2021 Davis RE et al. *JAMA Network Open.*

eTable 1. Model Classification Results of Best-Worst and Worst-Best Case Sensitivity Analyses.

Model	Model N	Sensitivity	Specificity	Correct Classification	AUC
**Model 1 (-SS)**					
Main	1087	58%	71%	61%	0.70
Best-Worst Case	1191	55%	75%	60%	0.72
Worst-Best Case	1191	56%	75%	60%	0.70
**Model 2 (+SS)**					
Main	1087	63%	69%	64%	0.72
Best-Worst Case	1191	61%	72%	64%	0.74
Worst-Best Case	1191	60%	74%	64%	0.73

* Cutoff: Probability=0.78 (maximum Youden Index)

© 2021 Davis RE et al. *JAMA Network Open.*

eTable 2. Missing Data Proportions and Social Risk Levels of Social Risk Score Component Variables.

	Total N=1203	Missing N=1203	Complete Data N=1087 (90%)
**Social Risk Score Components**	**n (%)**	**n (%)**	**n (%)**
**Marital status:**			
Never married, separated, divorced, widowed	485 (40%)	24 (2.0%)	432 (40%)
**Employment status:**			
Unemployed or disabled	278 (23%)	43 (3.6%)	266 (24%)
**Annual household income at CRC diagnosis:**			
200% FPL	689 (57%)	0 (0%)	614 (56%)
**Health insurance:**			
Medicaid or no insurance at cancer diagnosis	206 (17%)	8 (0.7%)	185 (17%)
**Health literacy:**			
Marginal or inadequate (‘often’ or ‘always’ on any D1)	168 (14%)	44 (3.7%)	149 (14%)
**Co-morbidities (in addition to CRC):**			
2 or more co-morbidities	544 (45%)	0 (0%)	494 (45%)
**Experiences of everyday discrimination:**			
Rarely, sometimes, often, or very often	404 (34%)	48 (4.0%)	381 (35%)
**Assists with personal care for another adult:**			
Provides care for at least one spouse, parent, parent-in-law, adult relative, or friend who lives in the participant’s home	161 (13%)	0 (0%)	152 (14%)
**Total Cumulative Risk, mean (SD)**	2.47 (1.61)	109 (9.1%)	2.46 (1.61)

© 2021 Davis RE et al. *JAMA Network Open.*

eTable 3. **Levels of Social Support Perceived From Different Sources (n=1,087)**

Source of Social Support	Amount of Support	
	Low[1]	High[2]
Family members (not including spouse or partner)	148 (14%)	939 (86%)
Friends	245 (23%)	842 (77%)
Health care providers	353 (32%)	734 (68%)
Spouse or partner	378 (35%)	709 (65%)
Members of religious community	561 (52%)	526 (48%)
Co-workers	756 (70%)	331 (30%)
Other people with CRC	851 (78%)	236 (22%)

[1] = Respondents answered “None,” “A little”, “Some,” “Does not apply”, or did not answer.
[2] = Respondents answered “Quite a bit” or “A lot.”

© 2021 Davis RE et al. *JAMA Network Open.*
